# Use of MRI to predict response following neoadjuvant chemotherapy for breast cancer: how accurately can it guide surgical choice?

**DOI:** 10.1186/bcr3771

**Published:** 2015-11-05

**Authors:** Sue Tan, Simon Lowes, Carol Ellen Holmes, Nidhi Sibal, Lesley McLean, Nerys Forester

**Affiliations:** 1 Newcastle Teaching Hospitals, Newcastle, UK

## Introduction

Breast MRI monitors tumour response to neoadjuvant chemotherapy (NAC) and guides breast-conserving-therapy (BCT). It is unclear how accurately MRI predicts pathological response. This audit investigates concordance between MRI findings and final pathology following NAC.

## Methods

Patients undergoing NAC between January 2011 and December 2014 were retrospectively identified. MRI was performed before, during and after NAC. At final MRI, response was graded as radiological complete response (CR no/<5 mm enhancement), partial response (PR <90% original enhancement), or no response (NR <10% reduction in enhancement). After surgery, pathological outcomes were no residual cancer (NRC), <5 mm invasive cancer/DCIS present (PRC), or >5 mm residual invasive cancer (RC). Radiological and pathological responses were either concordant or discordant.

## Results

Forty-six patients had NAC over 4 years (mean age 52 years), 43 IDC; three inflammatory carcinoma (not analysed). Radiological CR was seen in 19, PR in 18 and NR in six. Pathological outcome was NRC in 10, PRC in nine, and RC in 24. Responses were concordant in 30/43. BCT was attempted in 22 patients. Three required mastectomy for margins (despite two demonstrating radiological CR). MRI correctly predicted complete pathological response in 7/19 patients. In 12/19 there was residual disease despite MRI appearances. All six patients with no MRI response had residual invasive disease. Three patients with a partial MRI response demonstrated pathological complete response.

## Conclusion

MRI during NAC is useful, particularly when the MRI response is PR or NR. However, a complete radiological response predicts a complete pathological response in less than 50% of cases. Patients undergoing BCT following NAC should be aware of the risks of subsequent surgery.

